# Targeted therapy in CLL: changing the treatment paradigm

**DOI:** 10.18632/oncotarget.26964

**Published:** 2019-06-18

**Authors:** Christof Schneider, Daniela Steinbrecher, Stephan Stilgenbauer

**Affiliations:** ^1^ Department of Internal Medicine III, Ulm University Medical Center, Ulm, Germany; ^2^ Department of Internal Medicine I, Saarland University Medical Center, Homburg, Germany

**Keywords:** chronic lymphocytic leukemia, venetoclax, BCL2, deletion 17p, TP53

The therapeutic options for patients with CLL have developed remarkably within recent years, as several new drugs have paved the way to a chemotherapy–free treatment paradigm. A general feature of these drugs is their ability to induce cell death in a p53–independent manner, however their use is not restricted to CLL with defects in the TP53 pathway. The Bruton's tyrosine kinase (BTK) inhibitor ibrutinib has proven to be highly effective in both first–line and relapse. Results from the ILLUMINATE, Alliance North American Intergroup Study A041202 and ECOG–ACRIN E1912 trials show superiority of ibrutinib over varoius chemoimmunotherapy standard regimens in previously untreated CLL [1–3].

The orally available BCL–2 inhibitor venetoclaxcan induce apoptosis in a p53–independent manner, by displacing pro–apoptotic proteins like BIM and BAX from binding to BCL–2 and therefore inducing apoptosis. In CLL, which is universally characterized by high abundance of BCL–2, venetoclax seems to be the most effective single agent. Already the initial Phase 1 study showed substantial response rates and a manageable toxicity after adjustment to a dose escalation schedule [4]. The M13–982 study was a pivotal phase II trial with venetoclax in CLL patients harboring deletion of chromosome 17p (dell7p) [5]. The tumorsuppressor gene TP53 is located on chromosome 17p, and is recurrently mutated in CLL. In more than 80% of cases with dell7p the remaining allele is mutated, leading to an ineffective DNA damage response with impaired apoptosis. Patients with dell7p were found to have a more aggressive clinical course and inferior response to immuno–chemotherapy. As reported in the M13–982 trial, even in a relapsed I refractory setting with dell 7p, response rates of about80% can be achieved with single–agent venetoclax [5]. The drug is currently the most effective monotherapy achieving MRD negativity in peripheral blood in about30% of high risk cases. Additionally, it is well tolerated and discontinuation rates due to adverse events are low.

However, despite this high effectivity, patients candevelop secondary resistance to venetoclax over time with continuous drug administration. In the M13–982 trial the estimated duration of response at 24 months decreased to66% [5]. Specific mechanisms of resistance to venetoclax have been enigmatic, and just recently Blombery et al. demonstrated that the G101V mutation in BCL–2 confers acquired refractoriness by reducing the binding–affinity of venetoclax without disrupting the binding of pro­ apoptotic proteins to BCL–2 [6]. This mutation is mainly found in patients after long term exposure to venetoclax monotherapy [13]. In a proportion of venetoclax resistant CLL cases upregulation of other anti–apoptotic BCL–2 family members like BCL–XL have been shown to mediate resistance [6].

In order to minimize the risk for acquisition ofsecondary resistance, combination therapies and time limited treatment have been investigated. Based on the results of the phase III MURANO trial, the combination of venetoclax with rituximab has been licensed by FDA and EMA in patients with relapsed I refractory CLL irrespective of dell7p. In this trial venetoclax was given for a limited period of 2 years in combination with 6 administrations of Rituximab and compared to 6 cycles rituximab and bendamustine. The 24 months progression free survival estimates were 84.9% versus 36.3%, respectively, indicating that time limited combination therapies are also highly effective [7, 8]. Importantly, responses were durable after cessation of venetoclax, indicating that the deep responses were translating into prolonged survival times.

Current efforts in clinical trials are aiming to combine venetoclax with other highly effective novel drugs like obinutuzumab and ibrutinib in a time limited setting. In the CAPTIVATE trial the combination of ibrutinib and venetoclax in first–line therapy already achieved CR rates of 100% and MRD negativity in 82% [9]. The combination of obinutuzumab and veneteoclax have been shown to be well tolerated and highly effective in both first line and relapsed/refractory CLL [10]. Even in patients with co–existing conditions the combination was safe and improved progression free survival in comparison with the combination of obinutuzumab and chlorambucil [11]. In order to achieve even higher rates of MRD negativity and longer survival, triple combinations are currently tested. In a phase 1B study with relapsed I refractory CLL the combination of obinutuzumab, ibrutinib and venetoclax was well tolerated and achieved response rates of92% [12].

Due to the high rate of deep responses and long remission durations combined with good tolerability, venetoclax–based combination treatment approaches will most likely become a new standard in relapsed/refractory and front line CLL therapy. The fact that durable responses can be achieved with time limited treatment will not only reduce medical and financial toxicity, but also reduce the probability of resistance development.
